# Spatiotemporal delivery of multiple components of rhubarb-astragalus formula for the sysnergistic treatment of renal fibrosis

**DOI:** 10.3389/fphar.2024.1456721

**Published:** 2024-10-02

**Authors:** Qibin Zhang, Xiaofeng Ye, Lin Zhu, Zhishi Xu, Yu Hou, Qiaoying Ke, Jiawei Feng, Xiaowei Xie, Danfei Chen, Ji-Gang Piao, Yinghui Wei

**Affiliations:** ^1^ School of Pharmaceutical Sciences, Zhejiang Chinese Medical University, Hangzhou, China; ^2^ Department of Pediatrics, The First Affiliated Hospital of Zhejiang Chinese Medical University (Zhejiang Provincial Hospital of Chinese Medicine), Zhejiang Chinese Medical University, Hangzhou, China

**Keywords:** spatiotemporal delivery, multiple components, rhubarb, astragalus, multi-unit particulate system, renal fibrosis, gut microbiota

## Abstract

**Purpose:**

Rhubarb (*Rheum palmatum* L.) and astragalus (Radix astragali) find widespread used in clinical formulations for treating chronic kidney disease (CKD). Notably, the key active components, total rhubarb anthraquinone (TRA) and total astragalus saponin (TAS), exhibit superiority over rhubarb and astragalus in terms of their clear composition, stability, quality control, small dosage, and efficacy for disease treatment. Additionally, astragalus polysaccharides (APS) significantly contribute to the treatment of renal fibrosis by modulating the gut microbiota. However, due to differences in the biopharmaceutical properties of these components, achieving synergistic effects remains challenging. This study aims to develop combined pellets (CPs) and evaluate the potential effect on unilateral ureteral obstruction (UUO)-induced renal fibrosis.

**Methods:**

The CPs pellets were obtained by combining TRA/TAS-loaded SNEDDS pellets and APS-loaded pellets, prepared using the fluidized bed coating process. The prepared pellets underwent evaluation for morphology, bulk density, hardness, and flowing property. Moreover, the *in vitro* release of the payloads was evaluated with the CHP Type I method. Furthermore, the unilateral ureteral obstruction (UUO) model was utilized to investigate the potential effects of CPs pellets on renal fibrosis and their contribution to gut microbiota modulation.

**Results:**

The *ex-vivo* study demonstrated that the developed CPs pellets not only improved the dissolution of TRA and TAS but also delivered TRA/TAS and APS spatiotemporally to the appropriate site along the gastrointestinal tract. In an animal model of renal fibrosis (UUO rats), oral administration of the CPs ameliorated kidney histological pathology, reduced collagen deposition, and decreased the levels of inflammatory cytokines. The CPs also restored the disturbed gut microbiota induced by UUO surgery and protected the intestinal barrier.

**Conclusion:**

The developed CPs pellets represent a promising strategy for efficiently delivering active components in traditional Chinese medicine formulas, offering an effective approach for treating CKD.

## Graphical Abstract

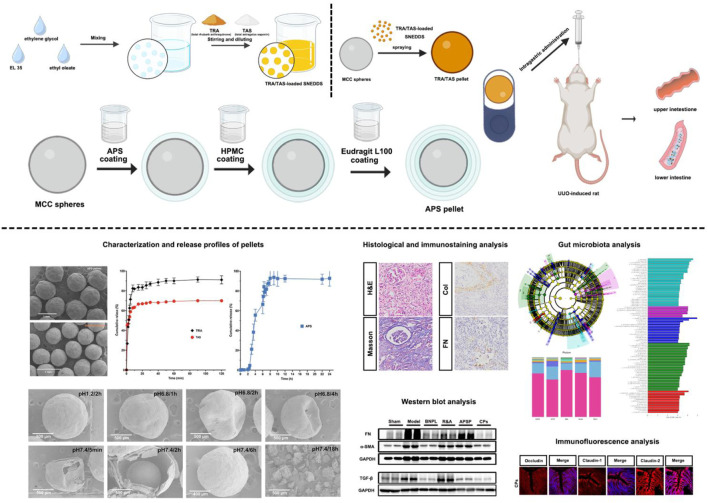

## 1 Introduction

Chronic kidney disease (CKD) is a global public health challenge associated with significant morbidity and mortality (Kalantar-Zadeh et al., 2021), imposing a substantial burden on individuals and society alike. Renal fibrosis, a prevalent pathway in CKD resulting in end-stage renal disease (ESRD), is characterized by renal interstitial fibroblast proliferation and extracellular matrix (ECM) accumulation, resulting in renal parenchyma damage and irreversible impairment of renal function (Huang et al., 2023; Nørregaard et al., 2023). Furthermore, renal fibrosis adversely affects not only the kidneys but also the heart (Panizo et al., 2021). Thus, halting renal fibrosis emerges as a promising strategy for preventing and treating CKD and ESRD. However, currently, there is a lack of direct treatment targeting renal fibrosis, except for the prevention of acute kidney disease transitioning to CKD or delaying CKD progression using angiotensin-converting enzyme inhibitors (ACEIs) and/or angiotensin receptor blockers (ARBs), as well as mineralocorticoid receptor blockers (Klinkhammer and Boor, 2023). Therefore, exploring effective approaches to impede the progression of renal fibrosis is imperative.

In recent years, clinical practice and studies have confirmed that traditional Chinese medicine (TCM), especially TCM formulas, have great potential in preventing and treating renal fibrosis (Liu X. Y. et al., 2022; Ma et al., 2023). Among them, rhubarb (*Rheum palmatum* L.) and astragalus (*Radix astragali*) are widely used in clinical formulations and have been reported to improve renal fibrosis in various ways (Gu et al., 2022; Yuan et al., 2020). Notably, a capsule containing pulverized rhubarb and astragalus, known as the rhubarb-astragalus capsule, has been clinically used for more than two decades to treat CKD, azotemia, and uremia (Zeng et al., 2020). Its potential mechanism of action may involve the regulation of the TGF-β1/Smad or NF-κB pathway (Adesso et al., 2018; Qin et al., 2021). Total rhubarb anthraquinone (TRA) and total astragalus saponin (TAS) are recognized as essential active components for kidney disease treatment (Wang et al., 2023b; Zhang et al., 2023). Researches indicate that anthraquinone in rhubarb attenuated renal fibrosis through inhibit the TGF-β/Smad, P38/MAPK, SIRT3/FOXO3/SOD2, PERK/ATF4/CHOP, P13K/Akt/mTOR signaling pathways (Wang et al., 2022). Sponins in astragalus were also reported to ameliorate renal fibrosis by blocking TGF-β/Smad signaling pathway (Zhou et al., 2017). Importantly, TRA and TAS offer advantages over rhubarb and astragalus due to their clear composition, stability, quality control, small dosage, and efficacy in disease treatment (Li A. P. et al., 2023; Xiang et al., 2020). Additionally, accumulating evidence suggests that CKD is associated with alterations in gut microbiota (Wang et al., 2023c; Zhou et al., 2022), which presents a promising novel target for slowing renal progression (Gharaie et al., 2023). Gut microbiota can be regulated through probiotics, prebiotics, synbiotics, fecal microbiota transplantation, and other interventions. Among prebiotics, various plant polysaccharides can increase the number and diversity of beneficial gut bacteria, significantly contributing to CKD treatment (Li A. Y. et al., 2023). For instance, the modification of gut microbiota using astragalus polysaccharides (APS) has been shown to attenuate kidney diseases (Guo et al., 2023; Peng et al., 2023; Zheng et al., 2021). Therefore, combining TRA, TAS and APS holds significant potential for renal fibrosis treatment. Nevertheless, overcoming the obstacle of inadequate water solubility in TRA and TAS is imperative to enhance their oral absorption (Cao et al., 2017; Zhou et al., 2023). To tackle this issue, promising approaches, such as prodrug strategies, salt formation, solid dispersion technology, and lipid-based delivery systems, are proposed (Bhujbal et al., 2021; Nora et al., 2022). Among lipid-based formulation, self-nanoemulsifying drug delivery system (SNEDDS) is popular for oral delivery due to their high solubilization capacity, high drug encapsulation, ability to transport drugs into the lymphatic system, and capacity to enhance bypass transport (Salawi, 2022). In the present study, TRA and TAS were formulated into SNEDDS to enhance their oral delivery. Moreover, the TRA/TAS-loaded SNEDDS were further solidified into pellets to improve the stability of the SNEDDS, increase drug loading, and facilitate their transformation (Sha et al., 2021).

Additionally, as non-starch polysaccharides, the effective delivery of most plant polysaccharides to the intestinal system is anticipated due to the absence of active carbohydrate enzymes necessary for their digestion in the gastrointestinal tract (Wang et al., 2023a). However, due to their high hydrophilicity, plant polysaccharides also exhibit high solubility and swelling in aqueous media (Bayer, 2023), which impedes their delivery to the lower gastrointestinal tract (GIT) and their effect on gut microbiota (Yang et al., 2022). To address this problem, colonic drug delivery systems was proved to be an advantageous approach (Shahdadi Sardou et al., 2022). Among the types of colonic drug delivery systems, the most marketed products are pH- dependent parallel systems. Nevertheless, combining the pH - and time-dependent methods allows for minimal drug release in the upper GIT and maximum drug release in the lower GIT. In addition, pellets, a type of multiunit dosage form, facilitate the targeted delivery of payloads to specific sites within the GIT and modify drug release (Kulkarni et al., 2022). Therefore, the APS was incorporated into colon-targeted pellets utilizing a combination of pH- and time-dependent systems.

Here, TRA/TAS-loaded self-nanoemulsifying pellets and APS-loaded, colonic site-specific pellets were developed respectively and combined to enable efficient release and absorption of TRA/TAS in the upper GIT, and specific delivery of APS to the lower GIT in a spatiotemporal manner. Initially, TRA/TAS-loaded SNEDDS were prepared based on a ternary phase diagram. These pellets were then solidified using the fluid-bed coating process. Subsequently, APS-loaded, colonic site-specific pellets were produced using sustained-release and enteric coating in a fluidized bed system. By encapsulating TRA/TAS pellets and APS pellets in a hard capsule shell at a mass ratio of 1:2, the *in vitro* release of the payloads was evaluated using a CHP Type I method. Finally, the therapeutic efficacy, as well as the regulation of gut microbiota of the combined pellets was tested on UUO rats.

## 2 Materials and methods

### 2.1 Materials and animals

TRA (≥50% purity) was purchased from Nanjing Shizhou Biotechnology Co., Ltd. (Nanjing, China). TAS (≥98% purity) and APS (≥90% purity) were provided by Chengdu Jintaihe Co., Ltd. (Chengdu, China). Microcrystalline cellulose (MCC) spheres (600–710 μm) were purchased from Hangzhou Gaocheng Biotech and Health Co., Ltd. (Hangzhou, China). Colloidal silicon dioxide was purchased from Shanghai Yuanju Biotechnology Co., Ltd. (Shanghai, China). Eudragit L100 (Opadry^®^ Enteric) and hydroxypropyl methylcellulose (HPMC) (Methocel™, E5LV) were obtained from Shanghai Colorcon Coating Technology Co., Ltd. (Shanghai, China).

PageRuler Prestained Protein Ladder were purchased from Thermo Fisher Scientific, Inc. (Waltham, MA, United States). Hematoxylin-Eosin (H&E) staining kit and Masson’s trichrome staining kit were purchased from Beijing M&C Gene Technology Co., Ltd. (Beijing, China). All enzyme-linked immunosorbent assay (ELISA) kits were purchased from Jiangsu Meimian Industrial Co., Ltd. (Yancheng, China). The other chemicals and solvents used in the study were of analytical reagent grade.

Thirty-six SD rats (200 ± 20 g) were provided by Shanghai SLAC Laboratory Animal Co., Ltd. (License No. SCXK 2017-0005, Shanghai, China).

### 2.2 Preparation of pellets

#### 2.2.1 Preparation of APS pellets

APS pellets were prepared using the solution layering technique in a fluidized bed system (Mini-Glatt, Glatt GmbH, Germany). Briefly, 2 g of APS was dissolved in 10 mL of water and sprayed onto 20 g of MCC spheres. The atomization pressure was set at 1.0 bar, inlet temperature at 50°C–55°C, and fluidized air velocity at 25 m^3^/h. A 20% (w/w) HPMC solution in water was then sprayed onto the drug-loaded pellets until a 20% weight gain was achieved. After the sustained-release coating, a subcoating with 20% Eudragit L100 suspensions was applied to achieve an additional 20% weight gain using the same system. Finally, the pellets were fluidized for an additional 30 min and then dried in a vacuum oven at 40°C for 2 h.

#### 2.2.2 Preparation of TRA/TAS pellets

Initially, a SNEDDS preconcentrate was prepared by mixing 21 g of EL35, 14 g of ethylene glycol, and 15 g of ethyl oleate, vortexed for 5 min, 315 mg of TRA and 441 mg of TAS (at a mass ratio of 5:7) were then added under stirring. After dilution with deionized water (five times), the nanoemulsion was obtained. Subsequently, 30 g of colloidal silicon dioxide was added to this liquid nanoemulsion, which was then sprayed onto 20 g of MCC spheres using the fluidized bed system with atomization pressure adjusted to 2.5 bar. Finally, the TRA/TAS pellets were dried in a vacuum oven at 40°C for 2 h.

### 2.3 Characterization of pellets

The overall shape of obtained pellets was investigated using stereoscopic microscope. Surface morphology of the pellets was also observed by using scanning electron microscope (SEM) (SU8010, HITACHI Company, Japan) after samples being coating with gold under condition of argon atmosphere. The bulk density of the prepared pellets was measured by pouring pellets in a graduated cylinder of 100 mL, and calculation involves dividing the weight of the pellets by the apparent volume in the cylinder, as described in CHP, method III. The angle of repose (AOR) of all the prepared pellets was determined by releasing an appropriate quantity of pellets onto the plane from a distance of 5 cm. Then, the height (h) and radius (r) of the pile from the base were measured and the AOR (θ) of the samples was calculated according to the following equation:

$$\text{AOR }\left(\theta \right)=\tan^{‐1}h/r$$

Three batches of pellets were measured in parallel.

### 2.4 *In vitro* drug release

Subsequently, the APS pellets and TRA/TAS pellets were encapsulated in size 000 gelatin capsules at a mass ratio of 1:2. Drug release from these formulations was assessed using a CHP type II (paddle) apparatus under controlled conditions with a temperature of 37°C ± 0.5°C and a stirring rate of 100 rpm. Initially, the pellets were exposed to 900 mL of a 0.1 M hydrochloric acid solution. After 2 h, the dissolution medium was switched to phosphate buffer (PBS) of pH 6.8 for an additional 4 h. Finally, the dissolution medium was changed to PBS with a pH of 7.4, and dissolution was carried out for a total duration of 24 h. Samples (3 mL) were withdrawn from different media and replaced with fresh medium. The contents of aloe-emodin, rhein, emodin, chrysophanol, and physcion (five representative components of TRA (Aichner and Ganzera, 2015), and Astragaloside IV (a representative component of TAS (Huang J. et al., 2018) were determined by a previously validated HPLC method in our lab (Hou et al., 2023). APS content was determined using the phenol-sulfuric acid method (Liu and Tan, 2022). All experiments were conducted in triplicate. Specifically, the similarity factor (*f*_*2*_) was used to evaluate the release synchronicity between TRA and TAS, which was calculated using the following equation:

$$f_{2}=50\log\left\{{\left[1+\frac{1}{n}\sum_{t}^{n}{W_{t}\left|R_{t}-T_{t}\right|}^{2}\right]}^{-0.5}\times 100\right\}$$

where n is the total sampling times; R_t_ and T_t_ are the release values of the referenced and the tested sample at time t, respectively; W_t_ is an optional weight factor.

### 2.5 UUO model and treatments

The UUO model was performed on male SD rats weighing 260–280 g, following our previously established protocol (Sun et al., 2022). In brief, after intraperitoneal injection of pentobarbital sodium at a dose of 50 mg/kg to induce anesthesia, the left ureter of the rats was exposed, isolated, and ligated using a 4/0 silk thread. The sham control rats underwent the same surgical procedure without left ureteral ligation. The UUO-induced renal fibrosis models were randomly divided into six groups, each consisting of six rats: 1) Sham Group: Sham rats received intragastric administration of saline at a dosage of 10 mL/kg. 2) Model Group: UUO rats received intragastric administration of saline at a dosage of 10 mg/mL. 3) BNPL (Benazepril) Group: UUO rats were administered benazepril hydrochloride intragastrically at a dose of 10 mL/kg. 4) R&A Group: UUO rats were administered a mixture of TRA/TAS suspension and APS solution intragastrically at doses equivalent to 50 mg/kg of TRA, 100 mg/kg of TAS, and 100 mg/kg of APS. 5) APSP Group: UUO rats were administered APS pellets intragastrically at a dose of 100 mg/kg. 6) Combined Pellets (CPs) Group: UUO rats were administered TRA/TAS pellets and APS pellets intragastrically at doses equivalent to 50 mg/kg of TRA, 100 mg/kg of TAS, and 100 mg/kg of APS, respectively.

The administration of different formulations continued daily for 21 days after the rat had fully recovered from surgery. On the 22nd day, fecal samples from all groups were collected and preserved at −80°C for subsequent gut microbiota analysis. Blood samples were collected from each rat. At the end of the experiment, the animals were anesthetized under CO_2_ asphyxiation and euthanized by cervical dislocation, and their left kidneys and colon tissues were collected and prepared for further studies.

### 2.6 Cytokine assays

Serum concentrations of IL-6, IL-10, D-LA, ET, PCS, IS, DAO, IFN-γ, TNF-α, and TMAO were determined using ELISA assay kits as described previously (Zhan et al., 2022). Briefly, standards and samples were added to the micro-ELISA strip-plate wells, whereupon they were incubated for 30 min at 37°C, followed by washing with washing buffer for five times. Afterward, the samples were further incubated with a streptavidin-horseradish peroxidase (HRP)-conjugated antibody specific for an additional 30 min. After addition of chromogen solution, incubation in the dark for 10 min at 37°C, the samples was measured on a microplate reader at 450 nm.

### 2.7 Histological and immunostaining analysis

For histological and immunostaining analysis, kidney and colon tissues were fixed with 4% buffered paraformaldehyde, embedded in paraffin, and subsequently sectioned into 4 µm thick slices. These sections were then subjected to staining with H&E and Masson’s trichrome. Additionally, serial paraffin-embedded kidney sections were allocated for immunohistochemical (IHC) analysis. In this process, the kidney sections were initially treated with 3% hydrogen peroxidase for 10 min at room temperature for blocking purposes. Following this step, the sections underwent a PBS wash and were then incubated overnight at 4°C with primary antibodies (diluted at 1:1000) targeting TGF-beta (ab179695, Abcam), collagen I (ab34710, Abcam), and fibronectin (ab2413, Abcam). These sections were then exposed to the corresponding secondary antibodies (diluted at 1:10000) for 60 min at 37°C. Furthermore, immunofluorescence (IF) analysis was conducted on colon tissues. To initiate this procedure, the paraffin-embedded colon sections were initially incubated overnight at 4°C with the appropriate primary antibodies targeting occludin (ab216327, Abcam), claudin-1 (ab15098, Abcam), and claudin-2 (ab15098, Abcam). Subsequently, these sections were exposed to secondary antibodies for 60 min at 37°C, and stained with nucleic acid stain DAPI. All of the histological, IHC, and IF-stained slides were analyzed using ImageJ analysis software (ImageJ 1.53a, United States) in a blinded fashion.

### 2.8 Western blot analysis

Up to 40 mg of kidney tissue underwent initial homogenization using RIPA lysis buffer (strong), followed by 10-min centrifugation (10,000 g, 4°C). The supernatant protein concentration was determined with the BCA protein assay kit. An equivalent amount of protein (30 mg) was then subjected to electrophoresis on a 6%–10% SDS-PAGE gel, and subsequently transferred to a polyvinylidene fluoride (PVDF) membrane. After blocking the membranes with protein-free rapid blocking buffer, for 15 min, the membranes were incubated with primary antibodies (diluted at 1:5000) against TGF-β (ab179695, Abcam), α-SMA (bs-10196R, Bioss), and fibronectin (ab2413, Abcam), respectively, overnight at 4°C. Following this, the membranes were then washed and incubated with horse radish peroxidase-conjugated goat anti-rabbit IgG antibodies (ab6721, Abcam) for 1 h at room temperature. The visualization of bands was achieved using an enhanced chemiluminescence detection system (QuickChemi 5200, Monad, China). The anti-GAPDH antibody (ab181602, Abcam) served as an internal loading control.

### 2.9 Gut microbiota analysis

Fresh fecal samples from the sham, model, R&A, APSP, and CPs groups of rats were collected using the abdominal compression method, promptly gathered with sterile tweezers, and immediately frozen at −80°C for later analysis. DNA was extracted from these samples using a cetyltrimethylammonium bromide (CTAB) buffer and was eluted in 50 µL of Elution buffer and stored at −80°C. PCR amplification involved a 25 µL reaction mixture with 25 ng of template DNA, 12.5 µL of PCR Premix, 2.5 µL of primers, and PCR-grade water. PCR products were verified using 2% agarose gel, purified with AMPure XT beads, and quantified using a Qubit ds DNA BR Assay kit. The 16S rDNA sequencing and subsequent bioinformatics analysis were conducted by LC-Bio Technology Co., Ltd (Hangzhou, China).

### 2.10 Statistical analysis

All data were presented as the mean ± standard deviation (SD). Statistical analysis was performed using GraphPad Prism (GraphPad Prism 9.0.0, United States) software. Unpaired t-test was applied to compare two groups in an unpaired design and one-way analysis of variance (ANOVA) was performed to compare the data among multiple groups, followed by Tukey’s *post hoc* test. **P < 0.01, *P < 0.05 were considered statistically significant.

## 3 Results and discussion

### 3.1 Preparation and characterization of pellets

The fluidized bed system was utilized to prepare APS and TRA/TAS pellets. Process parameters such as atomization pressure, inlet temperature, and fluidized air velocity were optimized during drug layering for APS pellets. Due to high sampling rates and APS solution concentration causing agglomeration, a sampling rate of 5 rpm and 20% (w/w) APS were used, achieving smooth, uniform layering with a 98.5% yield. After drug layering, pellets were coated with 20% (w/w) HPMC and 20% Eudragit L100 sequentially. The low-viscosity HPMC in the coating layer enhanced process feasibility and processing time, while ensuring controlled drug release at the target site (Wan et al., 2019). Eudragit L100 in the coating formulation helped prevent premature APS release in the upper GIT due to its pH sensitivity (Alghurabi et al., 2022). The combination of HPMC and Eudragit L100 ensured targeted location and sustained release of APS, thereby improving regulation on intestinal microbiota (Zhao et al., 2022).

**FIGURE 1:**
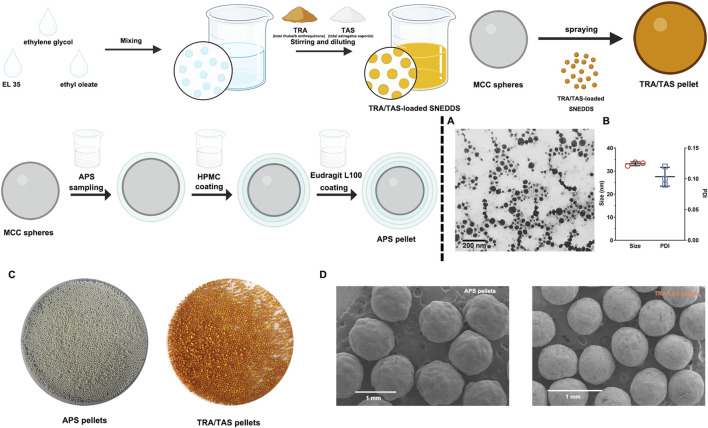
Characterizaion of APS pellets and TRA/TAS pellets. TEM images of the TRA/TAS-loaded SNEDDS **(A)**. Particle size and PDI of the TRA/TAS-loaded SNEDDS (n = 3) **(B)**. The photograph of APS pellets and TRA/TAS pellets **(C)**. SEM images of APS pellets and TRA/TAS pellets **(D)**.

Before preparation of TRA/TAS pellets, the optimization of the SNEDDS formulation was based on a ternary phase diagram. It was determined that the TRA/TAS ratio of EL35, ethylene glycol, and ethyl oleate at 21:14:15 resulted in clear, transparent, and uniform mixtures with particle size of (33.01 ± 0.12 nm) and a PDI of (0.10 ± 0.02) (Figure 1A). The contents of TRA and TAS in the SNEDDS formulation was 6.29 ± 0.07 mg/g and 8.80 ± 0.11 mg/g, respectively.

The prepared APS pellets appeared as white spheres, while the TRA/TAS pellets were yellow saffron spheres (Figure 1C). Under SEM, both APS pellets and TRA/TAS pellets displayed a spherical shape with a smooth surface (Figure 1D). Importantly, the TRA/TAS pellets had a notably smooth surface with no oil phase exudation, suggesting the effective solidification of the SNEDDS on the surface of MCC, as intended. In addition, the particle sizes of the two types of pellets were within the intervals of 750–1,000 μm. The bulk density of these pellets was nearly identical at 0.62 g/cm^3^, indicating their excellent filling into hard capsule shells (Tuyen et al., 2021). Moreover, the AOR of APS pellets and TRA/TAS pellets were 22.21 ± 0.19 and 23.75 ± 0.49, respectively, indicating promising flow properties of these pellets.

### 3.2 Release behavior of the pellets

**FIGURE 2:**
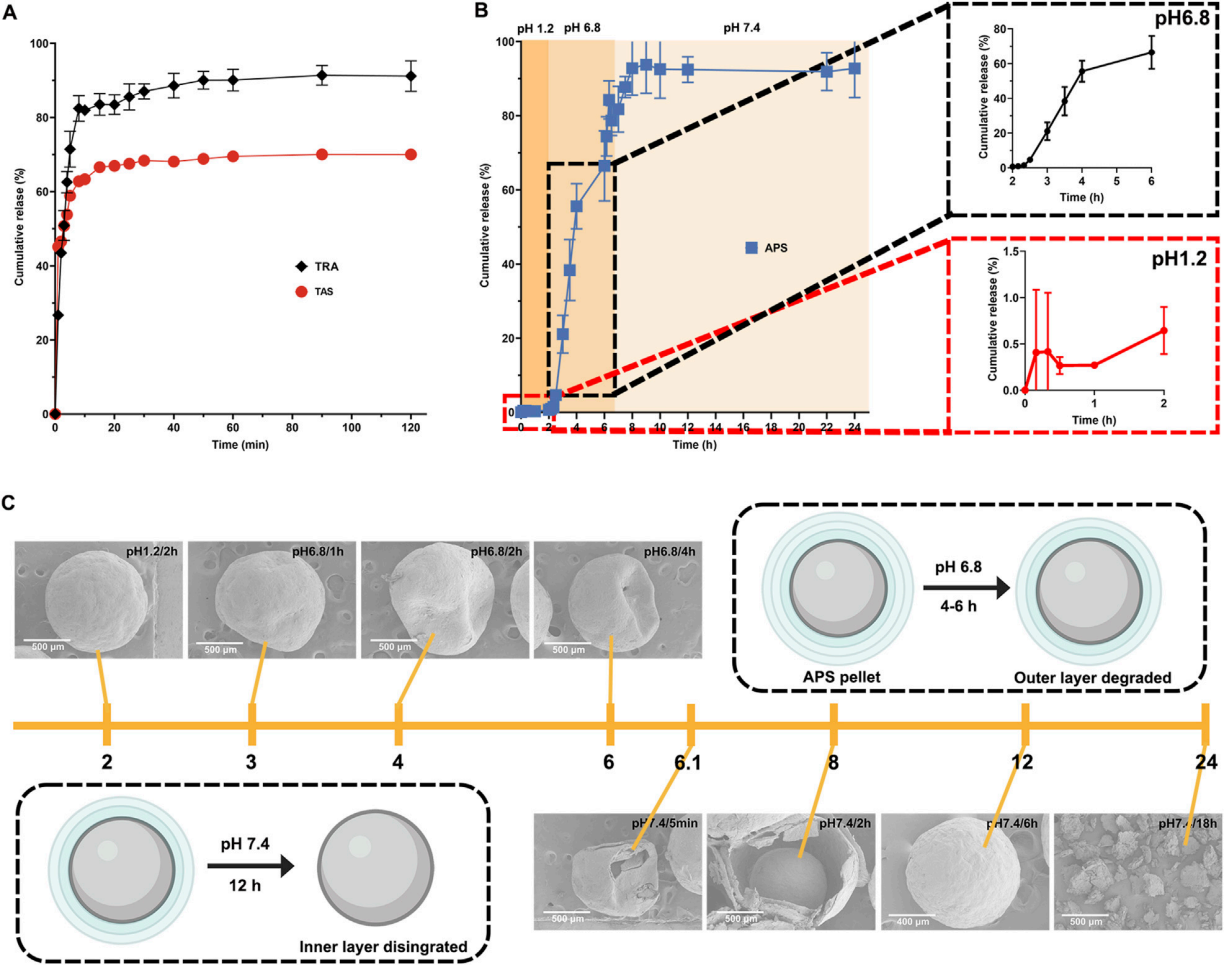
*In vitro* release profile of payloads from pellets. The release profiles of TRA and TAS in hydrochloric acid (pH1.2) in 2 h **(A)**. The release profile of APS in different media **(B)**. Structural evolution of APS pellets during *in vitro* dissolution in different media **(C)**.

In the current study, we assessed the *in vitro* release of TRA, TAS, and APS from pellets in three different pH media representative of the gastrointestinal tract. As depicted in Figure 2A, the release of TRA and TAS from the CPs pellets exceeded 80% and 60%, respectively, within the first 10 min in hydrochloric acid (0.1 M, pH 1.2). Conversely, no dissolution of raw TRA and raw TAS was observed, as both had limited solubility in water. This outcome signifies a significant increase in the solubility of TRA and TAS through the SNEDDS formulation. Notably, it was observed that the release of TAS is slightly inferior to that of TRA, which may due to its relative low solubility and stability issue in acid environment (Xu F. et al., 2023). Additionally, the calculated similarity factor (*f*_*2*_), a valuable parameter for evaluating the release synchronicity of multiple components (Chen et al., 2019), exceeded 50, indicating synchronous release of TRA and TAS from the CPs pellets (Song et al., 2019).

In contrast, as depicted in Figure 2B, almost no APS was released from the CPs pellets in hydrochloric acid (0.1 M, pH 1.2) within the initial 2 h. This suggests that the coating layer effectively prevented the entry of the acidic medium into the pellets, thereby avoiding the unwanted release of APS in the stomach. When exposed to PBS (pH 6.8), the outer coating layer (Eudragit L100) rapidly dissolved due to carboxylic group ionization at pH above 6.0 (Zu et al., 2021), resulting in APS release. Notably, APS release in PBS (pH 6.8) was relatively slow within the first hour and accelerated with cumulative release exceeding 70% within 4 h due to the complete breakdown of Eudragit L100 (Khan et al., 2022). Moreover, as pH increased from 6.8 to 7.4, APS release accelerated further, reaching a cumulative release of 95% within 24 h, primarily due to HPMC degradation. These findings confirm that the coating layer exhibits both pH-sensitive and time-dependent properties, preventing APS release in the upper gastrointestinal tract and ensuring its delivery to the lower intestine with subsequent gradual release (Shahdadi Sardou et al., 2022). Additionally, we observed the structural evolution of APS pellets in various media through SEM. As shown in Figure 2C, the APS pellets remained intact in hydrochloric acid (pH 1.2) for up to 2 h. However, exposure to PBS (pH 6.8) resulted in pellet breakage with visible pores on the surface for up to 4 h, indicating dissolution of the outer coating layer (Eudragit L100) and controlled drug release by the inner coating layer (Ibrahim and Alshora, 2021). Finally, when exposed to PBS (pH 7.4), pellet integrity was further compromised, and the pellets completely degraded over 18 h, leading to the complete release of APS, consistent with its release behavior.

Collectively, the rapid release of TRA and TAS in hydrochloric acid would facilitate their absorption in the upper small intestine. While the delayed release of APS in PBS provided by Eudragit L100 and HPMC could enable APS to reach the lower small intestine, as well as the colon. The different release patterns of TRA, TAS, and APS indicate their spatiotemporal delivery by the CPs, ensuring the improved bioavailability of TRA and TAS, colon-specific delivery of APS, which facilitates its regulation on gut microbiota.

### 3.3 Therapeutic efficacy of CPs pellets *in vivo*

#### 3.3.1 CPs pellets ameliorate the UUO-induced renal fibrosis

**FIGURE 3:**
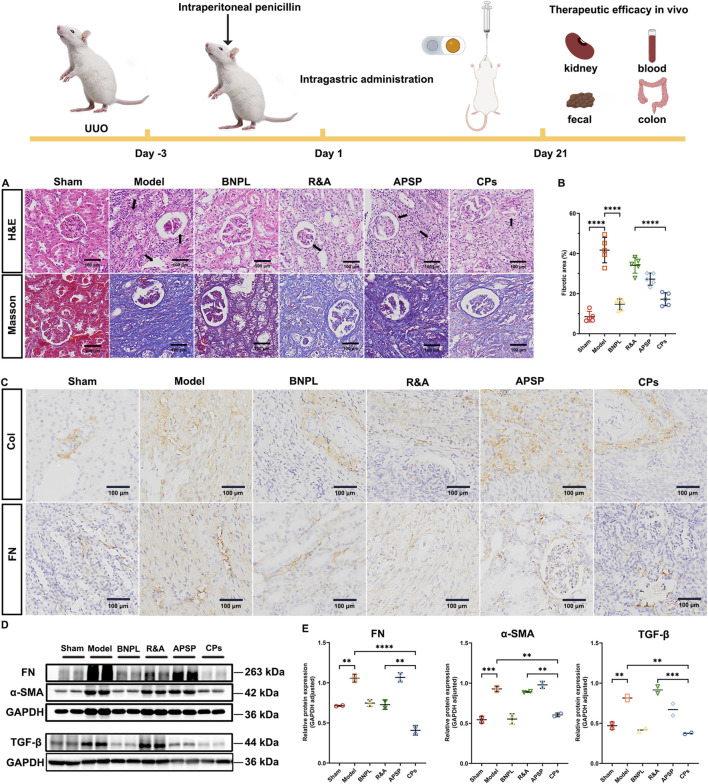
Pellets ameliorated UUO-induced renal fibrosis in rats. Representative images of H&E and Masson staining of kidney sections **(A)**. Quantification of Masson’s trichrome positive area of collagen-like matrix deposition (n = 5) **(B)**. Representative images of IHC staining of kidney sections **(C)**. Representative immunoblotting and densitometric analysis of FN, α-SMA and TGF-β in UUO rats’ kidney **(D, E)**.

Photomicrographs of H&E staining (Figure 3A) in the sham rats displayed intact glomeruli and tubules without any notable pathological changes. Conversely, severe pathological changes characterized by tubular dilation, tubular atrophy, and inflammatory cell infiltration was observed from model rats. Although the widening of the interstitial space with inflammatory cell infiltration was partially alleviated by the administration of R&A and APSP when compared to the model rats, adverse pathological changes, particularly tubular dilatation, remained visible. Importantly, all the pathological changes induced by UUO were significantly attenuated by the treatment with CPs pellets.

Additionally, collagen deposition in the tubulointerstitial area was assessed through Masson’s trichrome staining. As shown in Figures 3A, B, the sham rats showed almost no collagen deposition, while model rats exhibited significant collagen deposition with a fibrotic area of 41.6%. Notably, treatment with CPs pellets markedly ameliorated UUO-induced collagen deposition, achieving the lowest fibrotic area of 17.2% among all formulations. This suggests that CPs pellets exert potent anti-fibrotic effects in UUO rats.

Furthermore, we evaluated the expression of extracellular matrix components, collagen I (Col-I) and fibronectin (FN), in renal tissues through IHC staining (Figure 3C). UUO surgery led to a elevated expression of Col-I and FN, which was effectively ameliorated by the administration of CPs pellets. To further assess the regulation of ECM synthesis by CPs pellets, we determined the protein expression of FN and α-SMA in the obstructed kidney of rats using western blot analysis. As anticipated, the administration of CPs pellets significantly decreased the protein expression of FN and α-SMA (Figures 3D, E). Additionally, it has been reported that TGF-β plays a crucial role in the development of renal fibrosis (Park and Yoo, 2022). Upon binding to the type 2 TGF-β receptor (TβR2), TGF-β recruits TβR1, subsequently activating Smad2/3. This activation leads to an increase in the synthesis and expression of major collagen components within the extracellular matrix (ECM), including connective tissue growth factor, intercellular adhesion molecule-1, and type IV collagen fibers (Yuan et al., 2022). TGF-β can also regulate fibrosis-related genes through Smad-independent pathways, such as the activation of various mitogen-activated protein kinases (Hou et al., 2022). Therefore, it is imperative to restrain TGF-β signaling to impede the progression of renal fibrosis. Consequently, we assessed the expression of TGF-β in the UUO kidney and observed a significant decrease in TGF-β expression following the administration of CPs pellets (Figures 3D, E). In conclusion, while R&A and APSP have demonstrated anti-fibrotic activity against UUO surgery, the treatment with CPs pellets appears to enhance these therapeutic effects, potentially due to improved oral absorption facilitated by the SNEDDS and the prolonged retention of APS by the sustained-release coating layer.

#### 3.3.2 CPs pellets attenuate inflammation in UUO rats

**FIGURE 4:**
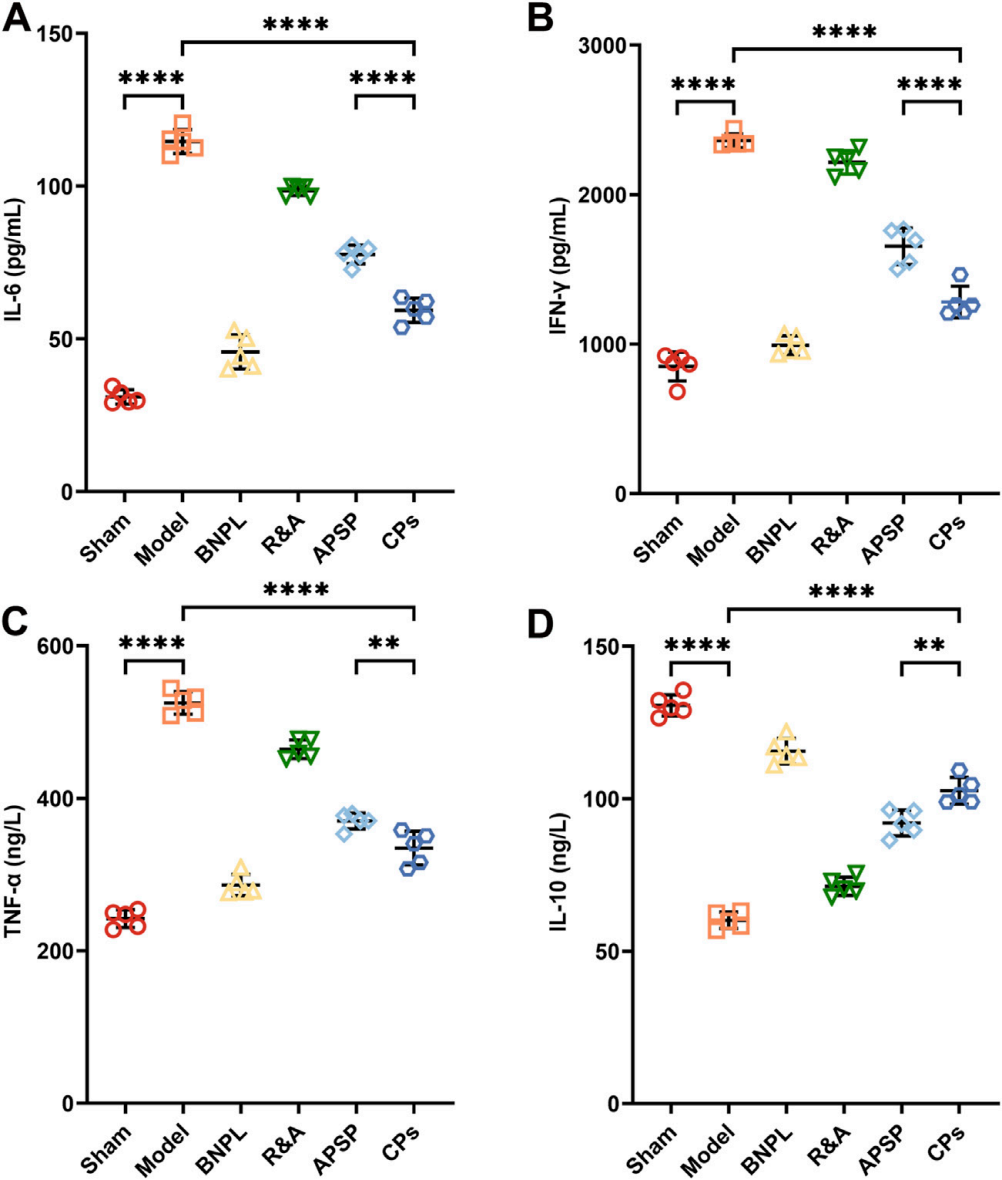
The levels of inflammatory cytokines IL-6 **(A)**, IFN-γ **(B)**, TNF-α **(C)**, and IL-10 **(D)** in serum from treated rats were measured by Elisa kits (n = 5).

Evidence suggests that renal inflammation plays a crucial role in the onset and progression of kidney diseases (Dhillon et al., 2023; Li et al., 2022). Inflammatory cells, including macrophages and T cells, release pro-inflammatory cytokines and chemokines, which activate fibroblasts and promote extracellular matrix protein deposition (Fu et al., 2022; Speer et al., 2022). Additionally, macrophage-derived inflammatory signaling molecules such as nuclear factor κB (NF-κB), tumor necrosis factor-α (TNF-α), TGF-β, interferon-γ (IFN-γ), and platelet-derived growth factor (PDGF) can exacerbate renal fibrosis (Cantero-Navarro et al., 2021). Conversely, anti-fibrotic factors like interleukin-10 (IL-10) (Jung et al., 2022) and bone morphogenetic protein-7 (BMP-7) are reportedly downregulated in kidney disease (Manzano-Lista et al., 2022; Peng et al., 2022). Therefore, serum levels of inflammatory signaling molecules, including IL-6, IFN-γ, TNF-α, and IL-10, were quantified using ELISA kits. Results (Figures 4A–D) showed significantly higher serum levels of IL-6, IFN-γ, and TNF-α and a decrease in IL-10 in model rats compared to sham rats. CPs pellet administration significantly suppressed the secretion of IL-6, IFN-γ, and TNF-α, and increased IL-10 secretion. These findings suggest that CPs pellets attenuated inflammation in UUO rats, corroborating the H&E staining results and contributing to renal fibrosis treatment.

### 3.4 Regulatory of gut microbiota by CPs pellets in UUO rats

**FIGURE 5:**
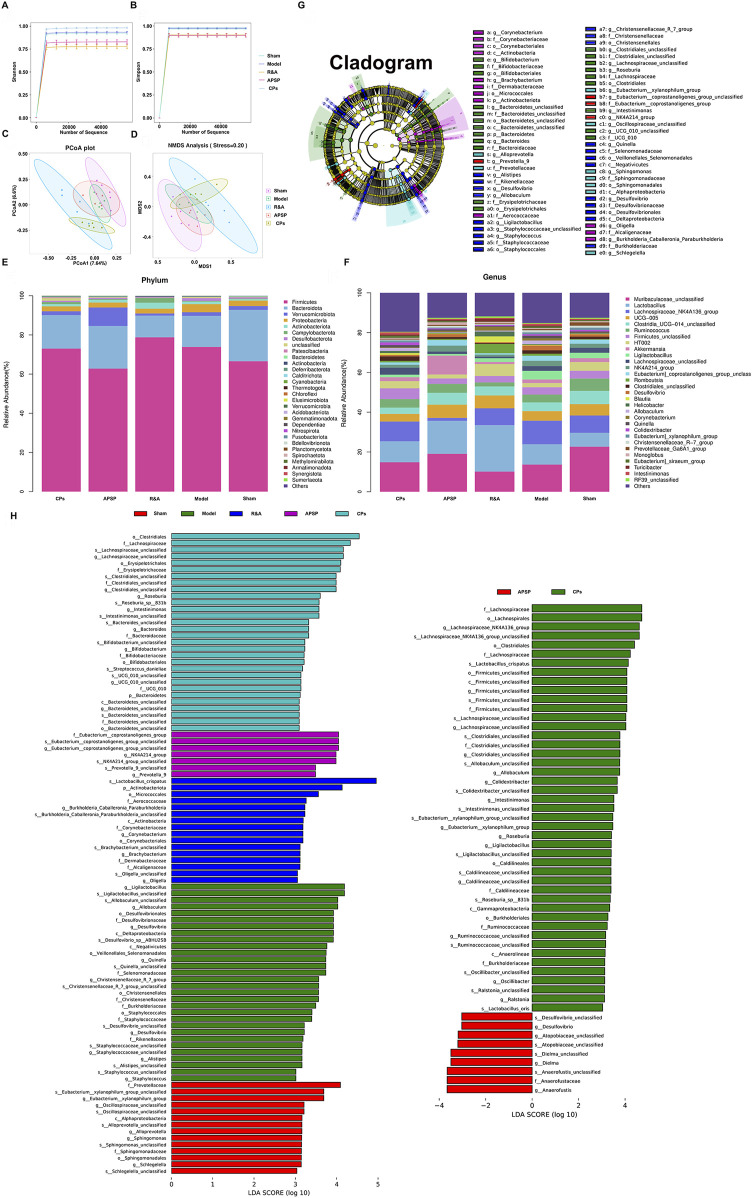
Pellets restored the intestinal microbial community composition and structure of UUO rats. α-Diversity in terms of Shannon index **(A)** and Simpson index **(B)**. PCoA **(C)** and NMDS **(D)** analyses of β-diversity based on OTUs. The cluster analysis of fecal flora among each group at the phylum level **(E)** and genus level **(F)**. LefSe analysis cladogram representing the significantly different taxas between different groups (LDA > 3, p < 0.05) **(G, H)**.

The impact of different formulations on the gut microbiota of UUO rats was assessed through the analysis of 16S rDNA amplicon sequencing of rat fecal samples. While no significant change was observed in community α-diversity among the various groups (Figures 5A, B), PCoA and NMDS analysis (Figures 5C, D) based on OTUs revealed a separation in gut microbiota composition between the model and sham groups. Interestingly, the gut microbiota composition of the CPs pellets and APSP groups also separated from the model group, indicating a restoration of gut microbiota from a UUO profile to a more normal profile.

At the phylum level, it was observed that the relative abundances of *Firmicutes* and *Proteobacteria* significantly increased in the model group, accompanied by a decrease in *Bacteroidetes* compared to the sham group. Because the dynamic balance and metabolic regulation of the gut microbiota are mainly dominated by *Firmicutes* and *Proteobacteria*, the changes in their abundance always affect the host’s metabolic processes. For instance, the increased *Proteobacteria* may be favorable to the growth of the *anaerobic Firmicutes*, thereby causing an imbalance in the gut microbiota (Bhargava et al., 2022; Li et al., 2020). Moreover, increased *Firmicutes* and *Proteobacteria* phyla have also been associated with elevated inflammatory responses in CKD patients, further impairing kidney function (Shahi et al., 2022). However, both the administration of CPs pellets and APSP significantly reduced the abundances of *Firmicutes* and *Proteobacteria* compared to the model group (Figure 5E), implying that APS may have a regulatory effect on the intestinal flora.

At the genus level (Figure 5F), a reduction in *Muribaculaceae_unclassified* and *UCG-005* was observed in the UUO group compared to the sham group. This reduction is known to lead to toxin accumulation in the intestine, the occurrence or exacerbation of gut inflammation, and a decrease in short-chain fatty acids (SCFAs) like butyrate (Lohia et al., 2022). Notably, both APSP and CPs pellets administration resulted in an increase in *Muribaculaceae_unclassified*, indicating that both formulations significantly modulated the dysbiosis of gut microbiota induced by UUO surgery. This suggests that the sustained-release pellets allowed for a prolonged release of APS in the lower GIT, enhancing its prebiotic function.

Furthermore, Linear discriminant analysis (LDA) effect size (LEfSe) analysis was conducted to identify dominant phylotypes that were significantly altered. Interestingly (Figures 5G, H), the microbial composition regulated by CPs pellets and APSP differed significantly. The administration of APSP specifically increased the abundance of *Anaerofustis*, while CPs pellets specifically enriched the abundances of specific genera, such as *Lachnospiraceae unclassified*. *Lachnospiraceae*, a bacterium that produces SCFAs, can protect the intestinal barrier by acidifying the intestinal environment and inhibiting the growth of harmful bacteria (Pan et al., 2022). The differences in microbial composition between the APSP and CPs pellet groups may be attributed to the coexistence of TRA and TAS in CPs pellets, particularly TAS, which could influence the gut microbiota to some extent (Zhou et al., 2021).

**FIGURE 6:**
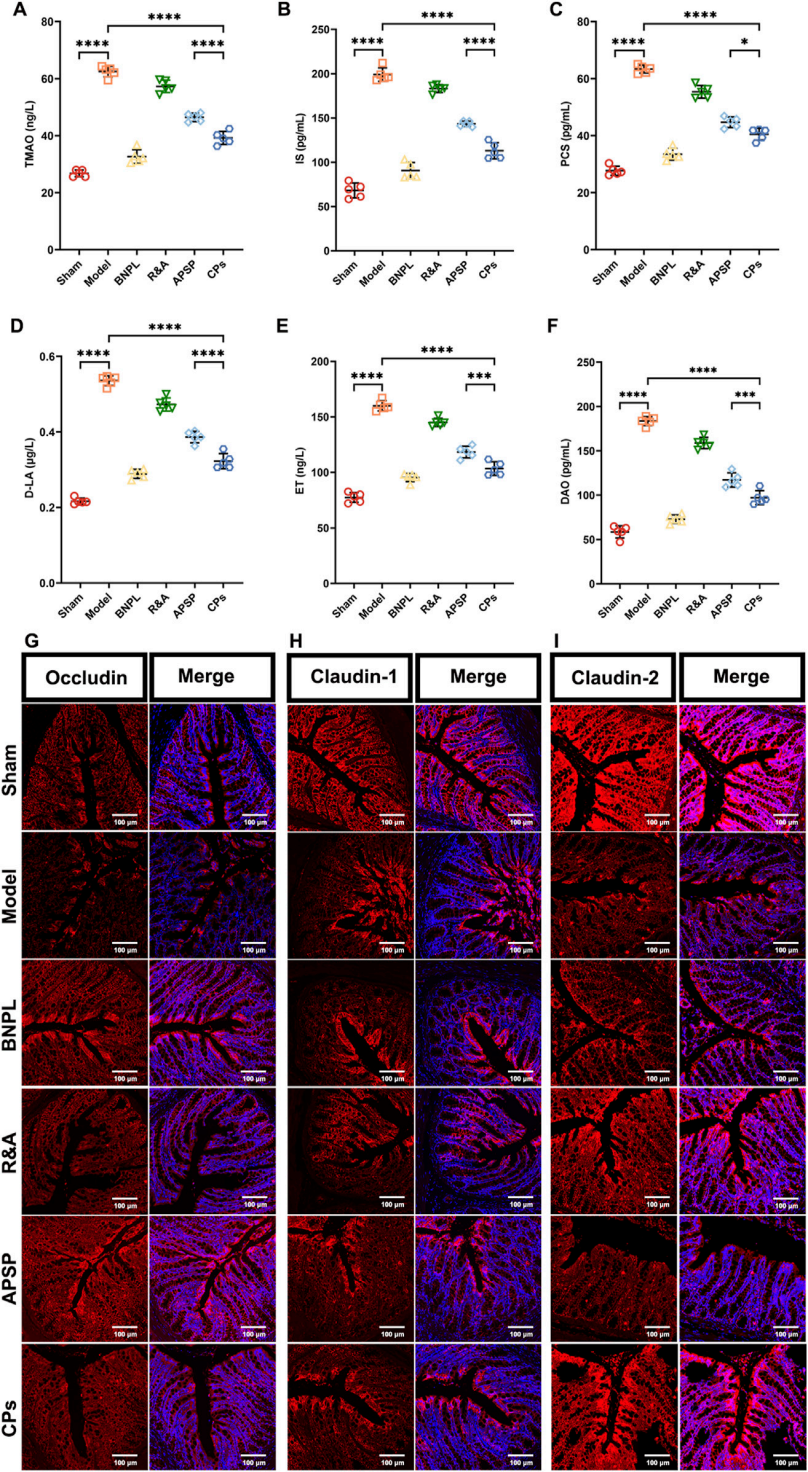
Pellets repaired the intestinal barrier. Serum levels of TMAO **(A)**, IS **(B)**, PCS **(C)**, D-LA **(D)**, ET **(E)** and DAO **(F)** (n = 5). Images of the Immunofluorescence-stained sections of Occludin **(G)**, Claudin-1 **(H)** and Claudin-2 **(I)** in colon tissue.

Additionally, it is known that gut microbiota dysbiosis induced by UUO surgery leads to increased endogenous toxin and disruption of the intestinal epithelial barrier, exacerbating kidney injury (Pan et al., 2023). Therefore, the serum levels of gut-derived endotoxins, including TMAO, IS, and PCS, as well as markers of intestinal permeability, including D-LA, ET, and DAO, were measured. As shown in Figures 6A–C, all formulations significantly reduced the serum levels of TMAO, IS, and PCS, with the most notable reductions observed in the APSP and CPs pellet groups. Similar effect of APSP and CPs pellets on the regulation of D-LA, ET, and DAO were also observed (Figures 6D–F). This indicates a more pronounced effect in restoring intestinal barrier function.

Finally, immunofluorescence staining was performed to assess the levels of occludin, claudin-1, and claudin-2, which are markers of tight junctions in colon tissue. As shown in Figures 6G–I, APSP, and CPs pellets exhibited significant protective effects on the intestinal mucosal barrier, consistent with previous reports (Liu J. Y. et al., 2022). Overall, APS played a vital role in the therapeutic efficacy of CPs pellets by regulating gut microbiota and protecting the intestinal barrier.

## 4 Conclusion

Promising combined pellets, containing TRA/TAS-loaded self-nanoemulsifying pellets and APS-loaded colonic site-specific pellets, were successfully developed for the synergistic treatment of renal fibrosis. The *ex-vivo* study demonstrated that the developed pellets could release TRA and TAS completely in the upper GIT and delivered APS specifically to the lower GIT. The *in vivo* study confirmed that combined pellets effectively mitigated UUO-induced renal fibrosis through spatiotemporal delivery of TRA/TAS and APS. The developed combined pellets could provide a promising strategy for effectively delivering multiple components of TCM formulas in a spatiotemporal manner, contributing to the synergistic treatment of chronic kidney disease. As a simple switchable drug delivery system, our system encourages further investigation on the use of MUPS platforms and spatiotemporal delivery of multiple components of TCM to different regions along the GIT.

## Data Availability

The raw data supporting the conclusions of this article will be made available by the authors, without undue reservation.

## References

[B1] Adesso S. Russo R. Quaroni A. Autore G. Marzocco S. (2018). Astragalus membranaceus extract attenuates inflammation and oxidative stress in intestinal epithelial cells via NF-κB activation and Nrf2 response. Int. J. Mol. Sci. 19, 800. 10.3390/ijms19030800 29534459 PMC5877661

[B2] Aichner D. Ganzera M. (2015). Analysis of anthraquinones in rhubarb (Rheum palmatum and Rheum officinale) by supercritical fluid chromatography. Talanta 144, 1239–1244. 10.1016/j.talanta.2015.08.011 26452953

[B3] Alghurabi H. Tagami T. Ogawa K. Ozeki T. (2022). Preparation, characterization and *in vitro* evaluation of Eudragit S100-coated bile salt-containing liposomes for oral colonic delivery of budesonide. Polymers 14, 2693. 10.3390/polym14132693 35808738 PMC9268925

[B4] Bayer I. S. (2023). Controlled drug release from nanoengineered polysaccharides. Pharmaceutics 15, 1364. 10.3390/pharmaceutics15051364 37242606 PMC10221078

[B5] Bhargava S. Merckelbach E. Noels H. Vohra A. Jankowski J. (2022). Homeostasis in the gut microbiota in chronic kidney disease. Toxins 14, 648. 10.3390/toxins14100648 36287917 PMC9610479

[B6] Bhujbal S. V. Mitra B. Jain U. Gong Y. Agrawal A. Karki S. (2021). Pharmaceutical amorphous solid dispersion: a review of manufacturing strategies. Acta Pharm. Sin. B 11, 2505–2536. 10.1016/j.apsb.2021.05.014 34522596 PMC8424289

[B7] Cantero-Navarro E. Rayego-Mateos S. Orejudo M. Tejedor-Santamaria L. Tejera-Muñoz A. Sanz A. B. (2021). Role of macrophages and related cytokines in kidney disease. Front. Med. 8, 688060. 10.3389/fmed.2021.688060 PMC8295566 34307414

[B8] Cao Y. J. Pu Z. J. Tang Y. P. Shen J. Chen Y. Y. Kang A. (2017). Advances in bio-active constituents, pharmacology and clinical applications of rhubarb. Chin. Med. 12, 36. 10.1186/s13020-017-0158-5 29299052 PMC5745730

[B9] Chen Z. J. Zhu Q. G. Qi J. P. Lu Y. Wu W. (2019). Sustained and controlled release of herbal medicines: the concept of synchronized release. Int. J. Pharm. 560, 116–125. 10.1016/j.ijpharm.2019.01.074 30753930

[B10] Dhillon P. Mulholland K. A. Hu H. L. Park J. W. Sheng X. Abedini A. (2023). Increased levels of endogenous retroviruses trigger fibroinflammation and play a role in kidney disease development. Nat. Commun. 14, 559. 10.1038/s41467-023-36212-w 36732547 PMC9895454

[B11] Fu Y. Xiang Y. Li H. L. Chen A. Q. Dong Z. (2022). Inflammation in kidney repair: mechanism and therapeutic potential. Pharmacol. Ther. 237, 108240. 10.1016/j.pharmthera.2022.108240 35803367

[B12] Gharaie S. Lee K. Newman-Rivera A. M. Xu J. J. Patel S. K. Gooya M. (2023). Microbiome modulation after severe acute kidney injury accelerates functional recovery and decreases kidney fibrosis. Kidney Int. 104, 470–491. 10.1016/j.kint.2023.03.024 37011727

[B13] Gu M. J. Zhou Y. F. Liao N. K. Wei Q. X. Bai Z. J. Bao N. (2022). Chrysophanol, a main anthraquinone from Rheum palmatum L. (rhubarb), protects against renal fibrosis by suppressing NKD2/NF-κB pathway. Phytomedicine 105, 154381. 10.1016/j.phymed.2022.154381 35988461

[B14] Guo M. F. Gao J. R. Jiang L. Dai Y. J. (2023). Astragalus polysaccharide ameliorates renal inflammatory responses in a diabetic nephropathy by suppressing the TLR4/NF-κB pathway. Drug Des. Devel. Ther. 17, 2107–2118. 10.2147/dddt.S411211 PMC10363349 37489175

[B15] Hou Y. Zhu L. Zhang Q. Ye X. Ke Q. Xu Z. (2023). Preparation and in-situ intestinal absorption study of Rhei Radix et Rhizoma-Astragali Radix components loaded self-microemulsion[in Chinese]. Chin. Tradit. Herb. Drugs 54, 3815–3823. 10.7501/j.issn.0253-2670.2023.12.008

[B16] Hou Y. L. Ding W. Y. Wu P. S. Liu C. Q. Ding L. N. Liu J. J. (2022). Adipose-derived stem cells alleviate liver injury induced by type 1 diabetes mellitus by inhibiting mitochondrial stress and attenuating inflammation. Stem Cell Res. Ther. 13, 132. 10.1186/s13287-022-02760-z 35365229 PMC8973806

[B17] Huang J. Yin L. Dong L. Quan H. Chen R. Hua S. (2018). Quality evaluation for Radix Astragali based on fingerprint, indicative components selection and QAMS. Biomed. Chromatogr. 32 (11), e4343. 10.1002/bmc.4343 30003570

[B18] Huang R. S. Fu P. Ma L. (2023). Kidney fibrosis: from mechanisms to therapeutic medicines. Signal Transduct. Target. Ther. 8, 129. 10.1038/s41392-023-01379-7 36932062 PMC10023808

[B19] Ibrahim M. A. Alshora D. H. (2021). Development and characterization of eudragit-RL-100-based aceclofenac sustained-release matrix pellets prepared via extrusion/spheronization. Polymers 13, 4034. 10.3390/polym13224034 34833333 PMC8624669

[B20] Jung K. Lee T. Kim J. Sung E. Song I. (2022). Interleukin-10 protects against ureteral obstruction-induced kidney fibrosis by suppressing endoplasmic reticulum stress and apoptosis. Int. J. Mol. Sci. 23, 10702. 10.3390/ijms231810702 36142626 PMC9504377

[B21] Kalantar-Zadeh K. Jafar T. H. Nitsch D. Neuen B. L. Perkovic V. (2021). Chronic kidney disease. Lancet 398, 786–802. 10.1016/s0140-6736(21)00519-5 34175022

[B22] Khan M. J. Huang W. C. Akhlaq M. Raza S. Hamadou A. H. Yuning G. (2022). Design, preparation, and evaluation of enteric coating formulation of HPMC and Eudragit L100 on carboxylated agarose hydrogel by using drug tartrazine. Biomed. Res. Int. 2022, 1042253. 10.1155/2022/1042253 35127935 PMC8816555

[B23] Klinkhammer B. M. Boor P. (2023). Kidney fibrosis: emerging diagnostic and therapeutic strategies. Mol. Asp. Med. 93, 101206. 10.1016/j.mam.2023.101206 37541106

[B24] Kulkarni N. Jain P. Shindikar A. Suryawanshi P. Thorat N. (2022). Advances in the colon-targeted chitosan based multiunit drug delivery systems for the treatment of inflammatory bowel disease. Carbohydr. Polym. 288, 119351. 10.1016/j.carbpol.2022.119351 35450623

[B25] Li A. P. Cui W. B. Zhao Y. R. Luo T. Zhang Q. Y. Liu Y. T. (2023). Exploration of the main effective constituent and the mechanism in Astragali Radix in the treatment for doxorubicin-induced nephropathy by integrating metabolomics and molecular docking. J. Ethnopharmacol. 305, 116074. 10.1016/j.jep.2022.116074 36577490

[B26] Li A. Y. Ding J. X. Shen T. Liang Y. Wei F. Wu Y. (2023). Radix paeoniae alba polysaccharide attenuates lipopolysaccharide-induced intestinal injury by regulating gut microbiota. Front. Microbiol. 13, 1064657. 10.3389/fmicb.2022.1064657 36713189 PMC9878331

[B27] Li L. Fu H. Y. Liu Y. H. (2022). The fibrogenic niche in kidney fibrosis: components and mechanisms. Nat. Rev. Nephrol. 18, 545–557. 10.1038/s41581-022-00590-z 35788561

[B28] Li W. Zhang Y. Mao W. Wang C. Yin S. (2020). Functional potential differences between Firmicutes and Proteobacteria in response to manure amendment in a reclaimed soil. Can. J. Microbiol. 66, 689–697. 10.1139/cjm-2020-0143 32717168

[B29] Liu B. Tan Z. (2022). Separation and purification of Astragalus membranaceus polysaccharides by deep eutectic solvents-based aqueous two-phase system. Molecules 27, 5288. 10.3390/molecules27165288 36014526 PMC9412596

[B30] Liu J. Y. Kong L. Z. Shao M. T. Sun C. H. Li C. X. Wang Y. Y. (2022). Seabuckthorn polysaccharide combined with astragalus polysaccharide ameliorate alcoholic fatty liver by regulating intestinal flora. Front. Endocrinol. 13, 1018557. 10.3389/fendo.2022.1018557 PMC9559367 36246879

[B31] Liu X. Y. Zhang X. B. Zhao Y. F. Qu K. Yu X. Y. (2022). Research progress of Chinese herbal medicine intervention in renal interstitial fibrosis. Front. Pharmacol. 13, 900491. 10.3389/fphar.2022.900491 35770077 PMC9235922

[B32] Lohia S. Vlahou A. Zoidakis J. (2022). Microbiome in chronic kidney disease (CKD): an omics perspective. Toxins 14, 176. 10.3390/toxins14030176 35324673 PMC8951538

[B33] Ma S. J. Zhao M. M. Chang M. Y. Shi X. J. Shi Y. Zhang Y. (2023). Effects and mechanisms of Chinese herbal medicine on IgA nephropathy. Phytomedicine 117, 154913. 10.1016/j.phymed.2023.154913 37307737

[B34] Manzano-Lista F. J. Sanz-Gómez M. González-Moreno D. Vega-Martín E. Gil-Ortega M. Schulz A. (2022). Imbalance in bone morphogenic proteins 2 and 7 is associated with renal and cardiovascular damage in chronic kidney disease. Int. J. Mol. Sci. 24, 40. 10.3390/ijms24010040 36613483 PMC9820638

[B35] Nora G.-I. Venkatasubramanian R. Strindberg S. Siqueira-Jørgensen S. D. Pagano L. Romanski F. S. (2022). Combining lipid based drug delivery and amorphous solid dispersions for improved oral drug absorption of a poorly water-soluble drug. J. Control Release 349, 206–212. 10.1016/j.jconrel.2022.06.057 35787914

[B36] Nørregaard R. Mutsaers H. A. Frøkiær J. Kwon T.-H. (2023). Obstructive nephropathy and molecular pathophysiology of renal interstitial fibrosis. Physiol. Rev. 103, 2827–2872. 10.1152/physrev.00027.2022 37440209 PMC10642920

[B37] Pan L. B. Yu H. Fu J. Hu J. C. Xu H. Zhang Z. W. (2023). Berberine ameliorates chronic kidney disease through inhibiting the production of gut-derived uremic toxins in the gut microbiota. Acta Pharm. Sin. B 13, 1537–1553. 10.1016/j.apsb.2022.12.010 37139409 PMC10149897

[B38] Pan X. Niu X. Y. Li Y. P. Yao Y. P. Han L. R. (2022). Preventive mechanism of lycopene on intestinal toxicity caused by cyclophosphamide chemotherapy in mice by regulating TLR4-MyD88/TRIF-TRAF6 signaling pathway and gut-liver Axis. Nutrients 14, 4467. 10.3390/nu14214467 36364730 PMC9655337

[B39] Panizo S. Martínez-Arias L. Alonso-Montes C. Cannata P. Martín-Carro B. Fernández-Martín J. L. (2021). Fibrosis in chronic kidney disease: pathogenesis and consequences. Int. J. Mol. Sci. 22, 408. 10.3390/ijms22010408 33401711 PMC7795409

[B40] Park C. H. Yoo T.-H. (2022). TGF-Β inhibitors for therapeutic management of kidney fibrosis. Pharmaceuticals 15, 1485. 10.3390/ph15121485 36558936 PMC9783223

[B41] Peng L. Zhang C. H. Xiao G. (2023). Astragalus polysaccharide alleviates angiotensin II-induced glomerular podocyte dysfunction by inhibiting the expression of RARRES1 and LCN2. Clin. Exp. Pharmacol. Physiol. 50, 504–515. 10.1111/1440-1681.13767 36876579

[B42] Peng W. Zhou X. C. Xu T. T. Mao Y. W. Zhang X. H. Liu H. M. (2022). BMP-7 ameliorates partial epithelial-mesenchymal transition by restoring SnoN protein level via Smad1/5 pathway in diabetic kidney disease. Cell Death Dis. 13, 254. 10.1038/s41419-022-04529-x 35314669 PMC8938433

[B43] Qin M. Y. Huang S. Q. Zou X. Q. Zhong X. B. Yang Y. F. Zhang Y. T. (2021). Drug-containing serum of rhubarb-astragalus capsule inhibits the epithelial-mesenchymal transformation of HK-2 by downregulating TGF-β1/p38MAPK/Smad2/3 pathway. J. Ethnopharmacol. 280, 114414. 10.1016/j.jep.2021.114414 34314804

[B44] Salawi A. (2022). Self-emulsifying drug delivery systems: a novel approach to deliver drugs. Drug Deliv. 29, 1811–1823. 10.1080/10717544.2022.2083724 35666090 PMC9176699

[B45] Sha K. Ma Q. F. Veroniaina H. Qi X. L. Qin J. Y. Wu Z. H. (2021). Formulation optimization of solid self-microemulsifying pellets for enhanced oral bioavailability of curcumin. Pharm. Dev. Technol. 26, 549–558. 10.1080/10837450.2021.1899203 33688786

[B46] Shahdadi Sardou H. Akhgari A. Mohammadpour A. H. Beheshti Namdar A. Kamali H. Jafarian A. H. (2022). Optimization study of combined enteric and time-dependent polymethacrylates as a coating for colon targeted delivery of 5-ASA pellets in rats with ulcerative colitis. Eur. J. Pharm. Sci. 168, 106072. 10.1016/j.ejps.2021.106072 34774715

[B47] Shahi S. K. Ghimire S. Lehman P. Mangalam A. K. (2022). Obesity induced gut dysbiosis contributes to disease severity in an animal model of multiple sclerosis. Front. Immunol. 13, 966417. 10.3389/fimmu.2022.966417 36164343 PMC9509138

[B48] Song L. J. Liang L. P. Shi X. Y. Chen H. L. Zhao S. M. Chen W. F. (2019). Optimizing pH-sensitive and time-dependent polymer formula of colonic pH-responsive pellets to achieve precise drug release. Asian J. Pharm. Sci. 14, 413–422. 10.1016/j.ajps.2018.05.012 32104470 PMC7032081

[B49] Speer T. Dimmeler S. Schunk S. J. Fliser D. Ridker P. M. (2022). Targeting innate immunity-driven inflammation in CKD and cardiovascular disease. Nat. Rev. Nephrol. 18, 762–778. 10.1038/s41581-022-00621-9 36064794

[B50] Sun J. Xu Z. S. Hou Y. Yao W. J. Fan X. D. Zheng H. S. (2022). Hierarchically structured microcapsules for oral delivery of emodin and tanshinone IIA to treat renal fibrosis. Int. J. Pharm. 616, 121490. 10.1016/j.ijpharm.2022.121490 35091004

[B51] Tuyen N. T. L. Nghiem L. Q. Tuan N. D. Le P. H. (2021). Development of a scalable process of film-coated bi-layer tablet containing sustained-release metoprolol succinate and immediate-release amlodipine besylate. Pharmaceutics 13, 1797. 10.3390/pharmaceutics13111797 34834212 PMC8618854

[B52] Wan D. W. Zhao M. Zhang J. J. Luan L. B. (2019). Development and *in vitro*-*in vivo* evaluation of a novel sustained-release loxoprofen pellet with double coating layer. Pharmaceutics 11, 260. 10.3390/pharmaceutics11060260 31195668 PMC6631012

[B53] Wang D. D. Wang W. B. Wang P. Wang C. Niu J. T. Liu Y. (2023a). Research progress of colon-targeted oral hydrogel system based on natural polysaccharides. Int. J. Pharm. 643, 123222. 10.1016/j.ijpharm.2023.123222 37454829

[B54] Wang H. Jiang Q. Kang L. Yuan L. Chen G. Cui X. X. (2023b). Rheum officinale and Salvia miltiorrhiza inhibit renal fibrosis via miR-21/PTEN/Akt signaling pathway *in vitro* and *in vivo* . J. Ethnopharmacol. 304, 115928. 10.1016/j.jep.2022.115928 36513264

[B55] Wang H. C. Ainiwaer A. Song Y. X. Qin L. Peng A. Bao H. (2023c). Perturbed gut microbiome and fecal and serum metabolomes are associated with chronic kidney disease severity. Microbiome 11, 3. 10.1186/s40168-022-01443-4 36624472 PMC9827681

[B57] Wang Y. Yu F. Li A. He Z. Qu C. He C. (2022). The progress and prospect of natural components in rhubarb (Rheum ribes L.) in the treatment of renal fibrosis. Front. Pharmacol. 13, 919967. 10.3389/fphar.2022.919967 36105187 PMC9465315

[B58] Xiang H. Zuo J. X. Guo F. Y. Dong D. S. (2020). What we already know about rhubarb: a comprehensive review. Chin. Med. 15, 88. 10.1186/s13020-020-00370-6 32863857 PMC7448319

[B59] Xu F. Li M. Que Z. Su M. Yao W. Zhang Y. (2023). Combined chemo-immuno-photothermal therapy based on ursolic acid/astragaloside IV-loaded hyaluronic acid-modified polydopamine nanomedicine inhibiting the growth and metastasis of non-small cell lung cancer. J. Mater Chem. B 11 (15), 3453–3472. 10.1039/d2tb02328h 37009696

[B61] Yang W. N. Zhao P. Li X. Guo L. P. Gao W. Y. (2022). The potential roles of natural plant polysaccharides in inflammatory bowel disease: a review. Carbohydr. Polym. 277, 118821. 10.1016/j.carbpol.2021.118821 34893238

[B62] Yuan H. Wu X. L. Wang X. M. Yuan C. F. (2020). Chinese herbal decoction astragalus and angelica exerts its therapeutic effect on renal interstitial fibrosis through the inhibition of MAPK, PI3K-Akt and TNF signaling pathways. Genes Dis. 9, 510–521. 10.1016/j.gendis.2020.06.001 35224164 PMC8843878

[B63] Yuan Q. Ren Q. Li L. Tan H. S. Lu M. Z. Tian Y. (2022). A Klotho-derived peptide protects against kidney fibrosis by targeting TGF-β signaling. Nat. Commun. 13, 438. 10.1038/s41467-022-28096-z 35064106 PMC8782923

[B64] Zeng X. Cai G. Z. Liang T. L. Li Q. Q. Yang Y. F. Zhong X. B. (2020). Rhubarb and Astragalus capsule attenuates renal interstitial fibrosis in rats with unilateral ureteral obstruction by alleviating apoptosis through regulating transforming growth factor beta1 (TGF-β1)/p38 mitogen-activated protein kinases (p38 MAPK) pathway. Med. Sci. Monit. 26, e920720. 10.12659/msm.920720 32205836 PMC7111584

[B65] Zhan X. Peng W. Wang Z. Liu X. Dai W. Mei Q. (2022). Polysaccharides from garlic protect against liver injury in DSS‐induced inflammatory bowel disease of mice via suppressing pyroptosis and oxidative damage. Oxid. Med. Cell. Longev. 2022, 2042163. 10.1155/2022/2042163 36017235 PMC9398839

[B66] Zhang F. Wu R. Liu Y. F. Dai S. Xue X. Y. Li Y. X. (2023). Nephroprotective and nephrotoxic effects of Rhubarb and their molecular mechanisms. Biomed. Pharmacother. 160, 114297. 10.1016/j.biopha.2023.114297 36716659

[B67] Zhao B. B. Du J. Zhang Y. Y. Gu Z. B. Li Z. F. Cheng L. (2022). Polysaccharide-coated porous starch-based oral carrier for paclitaxel: adsorption and sustained release in colon. Carbohydr. Polym. 291, 119571. 10.1016/j.carbpol.2022.119571 35698392

[B68] Zheng W. Huang T. Tang Q. Z. Li S. Qin J. Chen F. (2021). Astragalus polysaccharide reduces blood pressure, renal damage, and dysfunction through the TGF-β1-ILK pathway. Front. Pharmacol. 12, 706617. 10.3389/fphar.2021.706617 34690754 PMC8527034

[B69] Zhou J. X. Zhang N. H. Zhao L. Wu W. Zhang L. B. Zhou F. (2021). Astragalus polysaccharides and saponins alleviate liver injury and regulate gut microbiota in alcohol liver disease mice. Foods 10, 2688. 10.3390/foods10112688 34828972 PMC8623381

[B70] Zhou R. X. Guo T. K. Li J. L. (2023). Research progress on the antitumor effects of astragaloside IV. Eur. J. Pharmacol. 938, 175449. 10.1016/j.ejphar.2022.175449 36473596

[B71] Zhou W. Wu W. H. Si Z. L. Liu H. L. Wang H. Y. Jiang H. (2022). The gut microbe Bacteroides fragilis ameliorates renal fibrosis in mice. Nat. Commun. 13, 6081. 10.1038/s41467-022-33824-6 36241632 PMC9568537

[B72] Zhou X. Sun X. Gong X. Yang Y. Chen C. Shan G. (2017). Astragaloside IV from Astragalus membranaceus ameliorates renal interstitial fibrosis by inhibiting inflammation via TLR4/NF-кB *in vivo* and *in vitro* . Int. Immunopharmacol. 42, 18–24. 10.1016/j.intimp.2016.11.006 27855303

[B74] Zu M. H. Ma Y. Cannup B. Xie D. C. Jung Y. J. Zhang J. M. (2021). Oral delivery of natural active small molecules by polymeric nanoparticles for the treatment of inflammatory bowel diseases. Adv. Drug Deliv. Rev. 176, 113887. 10.1016/j.addr.2021.113887 34314785

